# Respiratory and Pleural Pathogens in Octogenarians Hospitalized with COVID-19: Impact of Secondary Bacterial Pneumonia on Day-5 SOFA and Mortality

**DOI:** 10.3390/microorganisms14010164

**Published:** 2026-01-12

**Authors:** Petrinela Daliu, Felix Bratosin, Ovidiu Rosca, Monica Licker, Elena Hogea, Livia Stanga, Camelia Vidita Gurban, Delia Muntean

**Affiliations:** 1 Doctoral School, “Victor Babes” University of Medicine and Pharmacy Timisoara, 300041 Timisoara, Romania; petrinela.daliu@umft.ro; 2 Department of Infectious Disease, “Victor Babes” University of Medicine and Pharmacy Timisoara, 300041 Timisoara, Romania; felix.bratosin@umft.ro (F.B.); ovidiu.rosca@umft.ro (O.R.); 3 Methodological and Infectious Diseases Research Center, Department of Infectious Diseases, “Victor Babes” University of Medicine and Pharmacy Timisoara, 300041 Timisoara, Romania; 4 Discipline of Microbiology, Multidisciplinary Research Center of Antimicrobial Resistance, Faculty of Medicine, “Victor Babes” University of Medicine and Pharmacy Timisoara, 300041 Timisoara, Romania; licker.monica@umft.ro (M.L.); hogea.elena@umft.ro (E.H.); muntean.delia@umft.ro (D.M.); 5 Microbiology Laboratory, “Pius Brinzeu” County Clinical Emergency Hospital, 300723 Timisoara, Romania; 6 Department IV Biochemistry and Pharmacology, Discipline of Biochemistry, Faculty of Medicine, “Victor Babes” University of Medicine and Pharmacy Timisoara, 300041 Timisoara, Romania; gurban.camelia@umft.ro

**Keywords:** COVID-19, aged, 80 and over, pneumonia, bacterial, drug resistance, microbial, respiratory tract infections/microbiology

## Abstract

Background and Objectives: Secondary bacterial infection drives poor outcomes in older adults with COVID-19, but age-specific microbiology and its interaction with severity scores are not well defined. We characterized respiratory and pleural pathogens, resistance profiles, and their impact on day-5 SOFA/APACHE II in octogenarians versus younger adults. Methods: We performed a retrospective cohort study of adults with RT-PCR-confirmed coronavirus disease 2019 (COVID-19) at a tertiary infectious diseases center (≥80 years, *n* = 152; <65 years, *n* = 327). Respiratory and pleural samples were processed according to EUCAST standards. Identification employed matrix-assisted laser desorption/ionization time-of-flight mass spectrometry (MALDI-TOF MS). Pathogen distributions, susceptibilities, and rates of superimposed pneumonia, empyema, and bacteremia were compared by age, and associations between secondary pneumonia, day-5 SOFA/APACHE II, and 28-day mortality were analyzed. Results: Sputum was obtained in 67.1% of older and 65.7% of younger adults, with numerically higher culture positivity in older patients (73.5% vs. 65.1%). Pathogen spectra were similar, dominated by *Streptococcus pneumoniae* (24.0% vs. 24.3%), methicillin-susceptible *Staphylococcus aureus* (MSSA) (18.7% vs. 20.7%), methicillin-resistant *Staphylococcus aureus* (MRSA) (9.3% vs. 6.4%), and *Klebsiella pneumoniae*, including extended-spectrum β-lactamase (ESBL)-producing strains. Empyema was more frequent in octogenarians (7.9% vs. 3.1%), and pleural cultures were usually positive. Meropenem retained 100% activity against ESBL-producing *K. pneumoniae* and *Pseudomonas* in both strata. In ≥80-year-olds, superimposed pneumonia was associated with higher day-5 SOFA (6.6 vs. 5.5) and APACHE II (24.3 vs. 21.0) scores and markedly increased 28-day mortality (37.5% vs. 9.8%). Conclusions: In octogenarians with COVID-19, secondary bacterial pneumonia and empyema are frequent, microbiologically similar to younger adults, and strongly amplify organ dysfunction and mortality even with largely preserved carbapenem susceptibility.

## 1. Introduction

Older adults continue to absorb a disproportionate share of the global COVID-19 burden: a recent systematic review calculated a pooled case fatality rate of 13.4% for people ≥ 80 years—tenfold higher than that of the general adult population [1]. Eastern European surveillance data confirm that advanced age, regional deprivation, and health-system capacity synergistically amplify mortality risk, with Romania occupying the highest quintile of excess deaths per 100,000 inhabitants in 2024 [2]. Age-related immune and endothelial changes—reduced T-cell diversity, heightened basal inflammation, and microvascular vulnerability—likely amplify COVID-19 severity in the very old. These biology of aging factors interact with frailty and multimorbidity, increasing the risk of respiratory failure and death [3,4,5].

Chronological age rarely acts in isolation. Nursing home outbreaks illustrate how frailty, cognitive impairment, and polypharmacy compound viral lethality: in one multicenter Spanish series, a Clinical Frailty Scale ≥ 6 nearly doubled 30-day mortality despite similar viral loads [6]. Superimposed bacterial or fungal pneumonia is another geriatric Achilles’ heel, affecting up to one-third of ICU-treated older adults and independently increasing both ventilator days and in-hospital deaths [7,8,9]. These intersecting biological and microbiological vulnerabilities mandate fast, accurate triage tools that transcend simple age cutoffs.

Intensive care and early warning scores capture complementary physiology. APACHE II integrates 12 acute physiologic variables, age, and chronic health points to yield a 0–71 composite reflecting global severity. SOFA (0–24) tracks six organ systems (respiratory, coagulation, liver, cardiovascular, CNS, and renal) and is sensitive to evolving organ dysfunction. CURB-65 (0–5) is pneumonia-focused (confusion, urea, respiratory rate, blood pressure, and age ≥ 65). NEWS2 (0–20) aggregates routine bedside vitals and oxygen requirements for early deterioration detection. While APACHE II and SOFA are widely used in ICU triage, CURB-65 and NEWS2 prioritize bedside simplicity on general wards

Cross-sectional Iranian data showed that an APACHE II > 18 predicted ICU mortality with 78% sensitivity [10], while Dutch investigators demonstrated that mean SOFA over the first 96 h outperformed admission-only values for 28-day survival discrimination [11]. However, both reports enrolled mixed-age cohorts, and subsequent work revealed calibration drift in the very old: a multinational analysis of 3882 patients ≥ 80 years noted that chronic organ dysfunction already contributes half of the baseline SOFA score, blunting its acuity signal [12]. Parallel evidence from Brazil suggested that APACHE II retained acceptable discrimination but required higher cutoffs (≥20) to preserve specificity in octogenarians [10].

Early warning scores designed for ward escalation—NEWS2, qCSI, and REMS—offer bedside simplicity. A 2024 Malaysian study reported that NEWS2 ≥ 6 predicted respiratory deterioration within 72 h with an AUC of 0.81 [13], while a Turkish cohort confirmed similar thresholds for ICU transfer [14]. Nonetheless, incorporation of anthropometric adjustments (age and BMI) appears to refine prognostic granularity: a prospective cohort showed that adding BMI ≥ 30 improved NEWS2 mortality AUC from 0.79 to 0.84 [15].

Community-acquired pneumonia tools (CURB-65 and PSI) have also been tested. A 2024 Chinese study comparing CURB-65 and PSI found that both predicted 30-day mortality, but CURB-65 underestimated risk in patients ≥ 80 years old when severe lymphopenia was present [16]. Adding oxygen saturation and respiratory support variables—components captured by the quick COVID-19 Severity Index (qCSI)—improved calibration across age strata [17]. Despite these refinements, accuracy remains modest in the very old, especially beyond the first 48 h of admission, when secondary infections muddy the clinical picture [18].

Our primary objective was to quantify how secondary bacterial respiratory infection and antibiotic resistance patterns modify the prognostic performance of day-5 SOFA and APACHE II in older adults (≥80 years) compared with younger adults (<65 years). Secondary objectives were to: (1) describe age-stratified pathogen distributions and first-line antimicrobial susceptibilities in respiratory and pleural cultures; (2) evaluate the joint effect of age group, secondary bacterial pneumonia or empyema, and high severity scores on ICU admission, length of stay, and 28-day mortality; and (3) assess the incremental predictive value of microbiological variables when added to conventional severity scores in prognostic models.

## 2. Materials and Methods

### 2.1. Legal and Ethical Considerations

This retrospective cohort study was designed to evaluate the predictive accuracy of clinical scores—APACHE II, CURB-65, SOFA, and NEWS2—at admission and five days after symptom onset in predicting severe COVID-19 outcomes in older adult patients aged 80 years and older. The study was conducted at the Victor Babeș Hospital of Infectious Diseases, Timisoara, affiliated with the Victor Babeș University of Medicine and Pharmacy, a tertiary care hospital, from March 2022 to June 2024. A control group of patients under 65 years old was included for comparative analysis. The study protocol was approved by the Institutional Review Board. Given the retrospective design and use of anonymized routinely collected clinical data, the Institutional Review Board approved the study and waived the requirement for individual informed consent, in accordance with institutional policy. All patients sign on admission a form that allows for data collection for future studies. The study adhered to the ethical standards of the Declaration of Helsinki, EU Good Clinical Practice Directives (2005/28/EC), and the International Council for Harmonisation of Technical Requirements for Pharmaceuticals for Human Use (ICH) guidelines. All patient data were anonymized to ensure confidentiality.

### 2.2. Inclusion and Exclusion Criteria

Inclusion criteria for the older adult group were patients aged 80 years and older with RT-PCR-confirmed coronavirus disease 2019 (COVID-19). For the control group, patients under 65 years old with a confirmed COVID-19 diagnosis were included. All participants required availability of data necessary for calculating the specified clinical scores at both time points. Exclusion criteria included patients aged 65–79 years, those with incomplete medical records lacking essential data for score calculation, patients who did not consent to participate, and those who were transferred from or to other facilities, making follow-up data collection unreliable.

Patients aged 65–79 years were excluded a priori to create two clearly separated age strata representing contrasting geriatric vulnerability (≥80 years) versus younger adult physiology (<65 years). This design reduces heterogeneity in frailty/comorbidity profiles within the comparator group and minimizes interpretive overlap with score components that incorporate age thresholds (CURB-65) or age-weighting (APACHE II).

### 2.3. Study Variables

Data were abstracted from the electronic medical record at admission (index time point) and on hospital day 5 (120 ± 12 h post-admission) and included demographics (age and sex), body mass index, residence (home vs. nursing home), smoking/alcohol status, vaccination status, medical history (Charlson Comorbidity Index components), and baseline clinical parameters (vital signs: respiratory rate, heart rate, systolic blood pressure, temperature, and oxygen saturation/oxygen supplementation; mental status; arterial blood gases, including PaO_2_/FiO_2_ and pH; and laboratory tests: complete blood count with differential, creatinine, urea/BUN, bilirubin, platelets, CRP, ferritin, D-dimer, IL-6, and procalcitonin). Vaccination was recorded as binary (any prior COVID-19 vaccination: yes/no); dose number, product type, and timing were not available.

Infections were classified as follows. Superimposed bacterial pneumonia was defined as new or progressive radiographic consolidation plus compatible clinical features and either (i) a positive respiratory culture (sputum or tracheal aspirate) for a respiratory pathogen or (ii) a procalcitonin rise compatible with bacterial infection in the opinion of the treating physician. Empyema required radiological evidence of pleural fluid with complex/septated features and either pus on thoracentesis or a positive pleural fluid culture. Bacteremia was defined as ≥1 blood culture positive for a clinically significant organism not considered a contaminant. Resistant pathogens were defined a priori as MRSA or ESBL-producing Enterobacterales. For prognostic analyses, we created binary variables for: (a) any secondary bacterial pneumonia, (b) empyema, (c) bacteremia, and (d) isolation of a resistant pathogen (MRSA and/or ESBL-producing *Klebsiella pneumoniae*).

The PICO framework was as follows. Population: adults hospitalized with RT-PCR-confirmed COVID-19 at a tertiary infectious diseases hospital, stratified into two age groups (≥80 years and <65 years). Exposures: day-5 SOFA and APACHE II scores dichotomized at data-driven optimal cut-offs, presence of secondary bacterial pneumonia or empyema, and isolation of resistant pathogens (methicillin-resistant *Staphylococcus aureus* or ESBL-producing Enterobacterales) from respiratory or pleural specimens. Comparators: patients in the opposite age group and/or without secondary infection or resistant isolates. Outcomes: ICU admission, need for invasive mechanical ventilation, ICU length of stay, and 28-day all-cause mortality.

Superimposed bacterial pneumonia was defined as new or progressive radiographic consolidation plus compatible clinical features (fever or hypothermia, purulent sputum, leukocytosis/leukopenia, or increasing oxygen requirement) together with either: (i) a positive respiratory culture (sputum or tracheal aspirate) yielding a clinically significant bacterial pathogen (culture-positive pneumonia) or (ii) culture-negative pneumonia supported by a procalcitonin rise above the institutional bacterial-infection threshold and initiation/escalation of antibacterial therapy by the treating team. For transparency, we report the proportion of pneumonia episodes that were culture-positive versus culture-negative and performed sensitivity analyses restricted to culture-positive episodes.

### 2.4. Definitions and Calculation of Scores

APACHE II (Acute Physiology and Chronic Health Evaluation II) is a severity-of-disease classification system that evaluates a patient’s risk of mortality based on 12 physiological measurements, age, and previous health conditions within the first 24 h of admission to an intensive care unit. CURB-65 is a clinical tool used to assess the severity of pneumonia and guide treatment decisions based on five criteria: confusion, urea level, respiratory rate, blood pressure, and age 65 or older. SOFA (Sequential Organ Failure Assessment) is used to track a patient’s status during their stay in the ICU and assess the extent and progression of organ dysfunction based on parameters that include respiratory, coagulation, liver, cardiovascular, central nervous system, and renal function. NEWS2 (National Early Warning Score 2) is an enhancement of the original NEWS, designed to improve the detection of clinical deterioration in patients through systematic monitoring of vital signs such as respiratory rate, oxygen saturation, temperature, systolic blood pressure, pulse rate, and level of consciousness.

APACHE II: Sum of (a) worst acute physiologic points within the first 24 h (temperature, mean arterial pressure, heart rate, respiratory rate, oxygenation [PaO_2_/FiO_2_ or A–a gradient], arterial pH, serum sodium, potassium, creatinine, hematocrit, WBC, and Glasgow Coma Scale); (b) age points; and (c) chronic health points (immunocompromise or organ insufficiency). CURB-65: One point each for confusion, urea > 7 mmol/L, respiratory rate ≥ 30/min, low blood pressure (SBP < 90 mmHg or DBP ≤ 60 mmHg), and age ≥ 65. SOFA: Six organs scored 0–4 using PaO_2_/FiO_2_, platelet count, bilirubin, vasopressor need/mean arterial pressure, Glasgow Coma Scale, and creatinine or urine output; total 0–24. NEWS2: Points assigned to respiratory rate, oxygen saturation (with/without supplemental oxygen), temperature, systolic blood pressure, heart rate, and level of consciousness; total 0–20. In this study, all scores were computed from admission values and hospital day 5 values (120 ± 12 h post-admission) using prespecified rules when multiple measurements were present (worst value selected for SOFA; contemporaneous set used for APACHE II).

Missing data handling was prespecified. For each score time point, if multiple measurements were available, we used the worst value for SOFA components and the contemporaneous physiologic set for APACHE II within the defined window. If a required component was unavailable within the window, we used the nearest value within ±24 h; if still unavailable, that score at that time point was treated as missing, and the patient was excluded from analyses requiring that score (complete-case approach; no imputation).

### 2.5. Microbiological Methods

Sputum and pleural fluid specimens were processed per EUCAST standards [19]. Sputum quality was screened using the Bartlett score. Aerobic cultures used 5% sheep blood, chocolate, and MacConkey agar; pleural fluid additionally underwent anaerobic culture (CDC anaerobe 5% sheep blood agar). Identification employed MALDI-TOF MS (Bruker Biotyper; Bruker Daltonics GmbH & Co. KG, Bremen, Germany). Antimicrobial susceptibility testing used EUCAST 2024 breakpoints via VITEK^®^ 2 and/or disk diffusion with routine quality control strains (e.g., *E. coli* ATCC^®^ 25922, *S. aureus* ATCC^®^ 29213). ESBL and MRSA were confirmed by EUCAST-recommended phenotypic methods. First-isolate susceptibilities were reported.

### 2.6. Statistical Analysis and Software

Statistical analysis was performed using SPSS Statistics version 26.0. Continuous variables are presented as means ± standard deviation (SD), and categorical variables are presented as frequencies and percentages. Comparisons between the older adult and control groups were made using the Mann–Whitney U test for continuous variables and the chi-square test for categorical variables. Receiver operating characteristic (ROC) curves were constructed to determine the predictive accuracy of the clinical scores, and the area under the curve (AUC), sensitivity, specificity, and optimal cutoff values were calculated. Kaplan–Meier survival analysis was performed to compare survival times between groups, and the log-rank test was used to assess statistical significance. Cox proportional hazards regression analysis was used to identify independent predictors of mortality. A *p*-value of less than 0.05 was considered statistically significant. Calibration was assessed by Brier score, calibration slope/intercept from logistic models, and Hosmer–Lemeshow goodness-of-fit (10 groups). Where indicated, bootstrap optimism correction (1000 resamples) was applied.

ChatGPT (GPT-4; OpenAI, San Francisco, CA, USA; accessed August–September 2025) was used solely to improve English grammar, wording, and readability of the manuscript text. Prompts explicitly instructed the model not to alter scientific meaning, numerical values, statistical results, or references. No patient-level or identifiable data were entered into the tool. All AI-suggested edits were independently reviewed, verified, and, where necessary, revised by the authors, who take full responsibility for the content. ChatGPT was not used for data analysis, statistical modeling, literature screening/selection, or interpretation of results.

## 3. Results

Table 1 compares baseline characteristics, infectious complications, and outcomes between older adults (≥80 years, *n* = 152) and younger adults (<65 years, *n* = 327) hospitalized with COVID-19. As expected, older adults had a markedly higher mean age (83.46 ± 2.92 vs. 52.37 ± 10.54 years, *p* < 0.001), were more often female (59.2% vs. 47.7%, *p* = 0.017), and had a lower mean BMI (25.13 ± 4.08 vs. 27.86 ± 5.12 kg/m^2^, *p* < 0.001). Current smoking was less frequent in older adults (14.5% vs. 23.9%, *p* = 0.026), while alcohol use and COVID-19 vaccination rates were similar between groups (alcohol 9.2% vs. 15.0%, *p* = 0.071; vaccination 54.6% vs. 53.8%, *p* = 0.865). A high comorbidity burden (CCI > 2) was substantially more common in older adults (62.5% vs. 28.1%, *p* < 0.001). The distribution of COVID-19 severity shifted towards more severe disease in the older group, with only 30.9% classified as mild compared to 57.5% in younger adults, and a higher proportion with severe/critical illness (30.3% vs. 14.1%). Older adults more frequently required ICU admission (22.4% vs. 8.6%, *p* < 0.001), oxygen supplementation (38.2% vs. 15.9%, *p* < 0.001), and invasive mechanical ventilation (15.1% vs. 5.5%, *p* < 0.001). Superimposed bacterial pneumonia (26.3% vs. 14.7%, *p* < 0.001), radiologically confirmed empyema (7.9% vs. 3.1%, *p* = 0.021), and blood culture-positive bacteremia (11.8% vs. 6.7%, *p* = 0.046) were also more frequent in older adults, who had a slightly longer ICU stay (median 8 (5–14) vs. 6 (4–11) days, *p* = 0.033) and a markedly higher 28-day all-cause mortality (17.1% vs. 3.1%, *p* < 0.001). When analyzed as a continuous measure, comorbidity burden remained substantially higher in octogenarians (CCI median [IQR] 4 (3–6) vs. 2 (1–3), *p* < 0.001), consistent with the higher prevalence of CCI > 2.

Table 2 summarizes respiratory and pleural microbiology and first-line antimicrobial susceptibilities in older versus younger adults. Sputum submission rates were similar (67.1% [102/152] in older vs. 65.7% [215/327] in younger adults, *p* = 0.79). Among positive cultures, the pathogen distribution was broadly comparable between age groups: *Streptococcus pneumoniae* accounted for around one-quarter of isolates (24.0% vs. 24.3%, *p* = 0.96), MSSA for approximately one-fifth (18.7% vs. 20.7%, *p* = 0.68), and MRSA for under 10% (9.3% vs. 6.4%, *p* = 0.32). ESBL-negative *Klebsiella pneumoniae* (16.0% vs. 18.6%, *p* = 0.61), ESBL-producing *Klebsiella* (6.7% vs. 5.0%, *p* = 0.55), *Pseudomonas aeruginosa* (12.0% vs. 12.9%, *p* = 0.85), and other Gram-negative bacilli (13.3% vs. 12.1%, *p* = 0.78) showed no significant age-related differences. Composite susceptibility of isolates indicated generally high activity of key agents in both groups: ceftriaxone susceptibility among *S. pneumoniae* and MSSA was 83% in older vs. 94% in younger adults, and levofloxacin susceptibility was 78% vs. 85%. For Gram-negative bacilli, piperacillin–tazobactam susceptibility was 71% vs. 75%, whereas meropenem retained 100% susceptibility in both age strata for ESBL-producing *Klebsiella* and *Pseudomonas* isolates.

Table 3 focuses on pleural fluid findings in patients with radiological empyema. Empyema was more frequent among older adults (7.9%, 12/152) than younger adults (3.1%, 10/327; *p* = 0.021). Diagnostic thoracentesis was performed in the vast majority of empyema cases in both groups (91.7% [11/12] in older vs. 90.0% [9/10] in younger adults, *p* = 0.88). Sterile pleural fluid was found in a minority of patients (18.2% [2/11] vs. 33.3% [3/9], *p* = 0.63), while positive pleural cultures predominated (81.8% [9/11] in older vs. 66.7% [6/9] in younger adults, *p* = 0.64). The microbiological profile was similar, with the *Streptococcus anginosus* group being the most common isolate (3 vs. 2 cases), followed by MRSA (2 vs. 1), ESBL-producing *K. pneumoniae* (2 vs. 1), *P. aeruginosa* (1 vs. 1), and mixed anaerobic flora (1 vs. 1). First-isolate antibiotic susceptibility remained excellent: vancomycin and meropenem showed 100% susceptibility in both age groups, while ceftriaxone susceptibility among *Streptococcus* and ESBL-negative *Klebsiella* was somewhat lower in older adults (67% vs. 83%). Clindamycin demonstrated 100% susceptibility among anaerobic isolates in both groups.

Table 4 presents day-5 SOFA and APACHE II scores, ICU admission, and 28-day mortality stratified by age group and presence of superimposed bacterial pneumonia. Among older adults (≥80 years), patients without superimposed pneumonia (*n* = 112) had a mean day-5 SOFA score of 5.5 ± 2.0 and APACHE II of 21.0 ± 6.1, with ICU admission in 10.7% and 28-day mortality of 9.8%. In contrast, those with superimposed pneumonia (*n* = 40) showed higher organ dysfunction and acute illness severity (SOFA 6.6 ± 2.2; APACHE II 24.3 ± 6.7), along with markedly increased ICU admission (55.0%) and mortality (37.5%). A similar pattern was observed in younger adults (<65 years): patients without pneumonia (*n* = 279) had lower day-5 SOFA (2.5 ± 1.3) and APACHE II scores (15.4 ± 4.7) and relatively low ICU admission (3.9%) and mortality (1.4%), whereas those with pneumonia (*n* = 48) had higher SOFA (3.5 ± 1.5) and APACHE II (17.8 ± 5.8) and substantially higher ICU admission (35.4%) and mortality (12.5%). Overall, the table illustrates a stepwise increase in severity scores, ICU utilization, and short-term mortality associated with both older age and the presence of superimposed bacterial pneumonia.

Five days after symptom onset, older adult patients continued to show worse clinical parameters compared to the control group. Oxygen saturation levels further decreased in the older adult group (89.72 ± 4.57% vs. 93.12 ± 3.14%, *p* < 0.001), and the respiratory rate increased (24.16 ± 4.89 breaths per minute vs. 20.14 ± 3.92 breaths per minute, *p* < 0.001). The APACHE II score increased in the older adult group (21.85 ± 6.42 vs. 15.78 ± 5.14, *p* < 0.001), as did the SOFA score (5.47 ± 2.18 vs. 2.64 ± 1.32, *p* < 0.001). Table 5 presents these findings.

**Figure 1:**
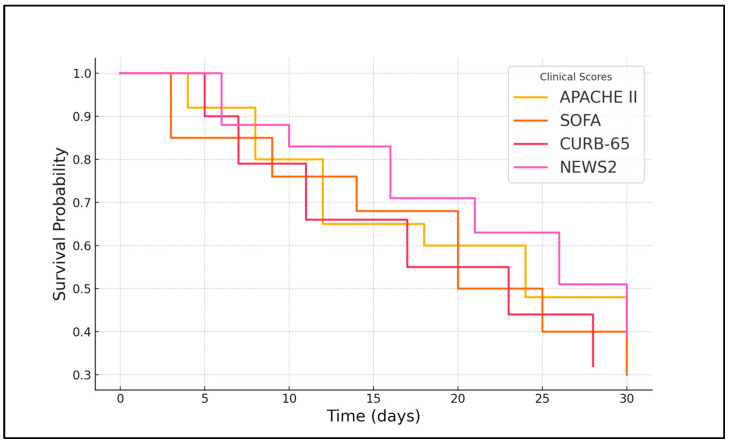
Kaplan–Meier analysis for severe COVID-19 development in older adult patients.

At admission, an APACHE II score greater than 19.50 was significantly associated with an increased risk of mortality, with a hazard ratio (HR) of 1.89 (95% CI: 1.12–3.18, *p* = 0.017). The SOFA score showed an even stronger association, with a hazard ratio of 2.72 (95% CI: 1.85–4.02, *p* < 0.001), indicating that organ dysfunction at admission was a significant predictor of death in this population. However, the CURB-65 score above 2.50 did not reach statistical significance (*p* = 0.061), and the NEWS2 score was not significantly associated with mortality at admission, with an HR of 1.20 (95% CI: 0.85–1.69, *p* = 0.299), as seen in Figure 1.

**Figure 2:**
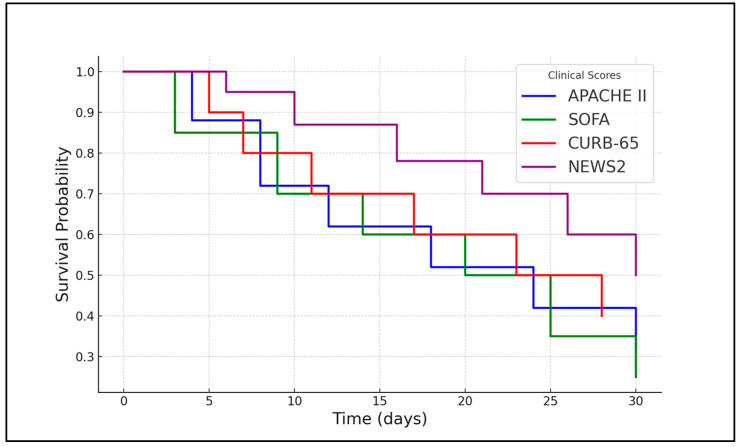
Kaplan–Meier analysis for mortality in older adult patients over 80 years old after COVID-19.

At five days post-symptom onset, the SOFA score remained the most robust predictor of mortality, with an HR of 3.10 (95% CI: 2.05–4.70, *p* < 0.001), suggesting that worsening organ function over time strongly correlates with death in older adult patients. APACHE II scores above 21.00 at this time point also indicated a significant increase in mortality risk, with an HR of 2.15 (95% CI: 1.42–3.25, *p* = 0.001). In contrast, neither CURB-65 (*p* = 0.181) nor NEWS2 (*p* = 0.652) scores at five days post-symptom onset showed significant predictive value for mortality in this cohort, as presented in Table 6 and Figure 2.

Superimposed bacterial pneumonia complicated the course of more than a quarter of older adult admissions (26.3%) versus 14.7% in younger patients (*p* < 0.001), mirroring higher sputum-culture positivity. Empyema echoed that pattern (7.9% vs. 3.1%, *p* = 0.021) and contributed to the greater incidence of culture-proven bacteremia in older adults (11.8% vs. 6.7%, *p* = 0.046). These infectious complications lengthened ICU stays (median 8 days, IQR (5–14) vs. 6 (4–11), *p* = 0.033) and dovetailed with a sharply elevated 28-day mortality of 17.1% compared with 3.1% in controls (*p* < 0.001) (Table 7).

In prespecified subgroup analyses, day 5 SOFA remained the most discriminative score in both octogenarians and younger adults, with lower optimal thresholds in the <65 cohort. Among patients ≥ 80 years, day 5 SOFA ≥ 4.85 achieved the highest AUC (0.854) and was independently associated with severe disease (HR 3.26, 95% CI 2.19–4.84) and 28-day mortality (HR 3.10, 95% CI 2.05–4.70). In patients < 65 years, day 5 SOFA ≥ 3.50 yielded an AUC of 0.828 and mortality HR of 2.42 (95% CI 1.56–3.75), as presented in Table 8.

To improve bedside interpretability, we calculated empiric adequacy for commonly used agents across culture-positive episodes (Table 9). Inadequacy of ceftriaxone- or fluoroquinolone-based strategies was primarily driven by MRSA, ESBL-producing *K. pneumoniae*, and Gram-negative non-susceptibility patterns, whereas carbapenem-based coverage remained highly reliable in both age strata.

In exploratory models restricted to culture-positive episodes (Table 10), higher illness severity and healthcare-associated context variables (ICU admission and nursing home residence) showed the strongest associations with empiric inadequacy of ceftriaxone or levofloxacin, supporting their potential utility as bedside surrogates for resistant or non-covered pathogens when culture data are pending (Table 10).

To directly test whether the association between superimposed bacterial pneumonia and outcomes differed by age stratum, we fit regression models including an age group × pneumonia interaction term (Table 11). On the multiplicative scale, the interaction was not statistically significant for 28-day mortality (interaction *p* = 0.47) or ICU admission (interaction *p* = 0.66), indicating that pneumonia increased risk in both age groups without strong evidence of differential relative effect. On the absolute scale, the excess mortality associated with pneumonia was larger in octogenarians (37.5% vs. 9.8%; risk difference 27.7%) than in younger adults (12.5% vs. 1.4%; risk difference 11.1%), supporting a greater absolute clinical impact in the very old.

## 4. Discussion

### 4.1. Analysis of Findings

These data support a microbiology-driven approach to older adults with COVID-19, in which early, good-quality respiratory sampling and systematic investigation of pleural collections are routine rather than selective. The high frequency of culture-proven secondary pneumonia (26.3%) and empyema (7.9%) in octogenarians, combined with the strong association of superinfection with higher day-5 SOFA/APACHE II and fourfold higher 28-day mortality, argues for low thresholds for sputum collection, blood cultures, and imaging-guided thoracentesis. Similar pathogen spectra across age groups suggest that empiric regimens can be aligned between older and younger adults, but the presence of MRSA and ESBL-producing Klebsiella mandates that local resistance data inform initial coverage. The preserved meropenem susceptibility supports its role as a dependable escalation option to be used within antimicrobial stewardship frameworks. Integrating microbiological results with dynamic SOFA trajectories can refine decisions on de-escalation, narrowing therapy once susceptibilities are known, while still protecting very old, high-risk patients from undertreatment. The empiric adequacy analysis translates isolate-level susceptibility into episode-level bedside probabilities, which is more clinically actionable than reporting susceptibilities by organism alone. This approach clarifies how frequently a given empiric regimen would be expected to cover the likely bacterial pathogen(s) in secondary pneumonia/empyema during COVID-19 hospitalizations [20].

In a similar manner, the study by Vermeiren et al. [21] found that the 90-day survival rate of older adult patients in the ICU during the first and second waves of COVID-19 in Belgium remained consistent, with 52.27% surviving in the first wave and 52.17% in the second, despite changes in treatment protocols such as the introduction of dexamethasone and increased dosing of prophylactic anticoagulation. These findings were echoed by the COVIP study led by Jung et al. [22], which included a broader European cohort and showed comparable survival rates during the initial 15 days of both surges. However, unlike the Belgian study, Jung et al. [22] reported a significant decline in survival after the first 15 days during the second surge, with 30-day and 90-day survival rates decreasing from 57% to 50% and from 51% to 40%, respectively. This discrepancy in outcomes highlights the potential impact of evolving treatment strategies and clinical practices on the survival of critically ill older adult patients.

Moreover, a study by López-Izquierdo et al. [23] investigated the effectiveness of established severity scores and developed a new predictive model for in-hospital mortality among older adult patients with COVID-19 pneumonia. They recorded an AUC of 0.81 for their new model, which was higher than the AUCs for the Pneumonia Severity Index (PSI), CURB-65, and Severe Community-Acquired Pneumonia (SCAP) scores, which showed moderate predictive power with AUCs of 0.74, 0.71, and 0.72, respectively. These findings align with the results of the study by Wang et al. [24], which evaluated the Modified Early Warning Score (MEWS) and found it comparably effective to other scores such as APACHE II, SOFA, and qSOFA for predicting in-hospital mortality in older adult COVID-19 patients. However, MEWS demonstrated superior predictive accuracy over SIRS and CURB-65, with significant differences in AUCs, highlighting the potential of MEWS as a rapid assessment tool in this high-risk population.

A study by Yong Sub Na et al. [25] highlighted the effectiveness of various scoring systems, such as the SOFA and CFS scores, for predicting in-hospital mortality in older adult patients with severe COVID-19. The study found that the SOFA score had a high predictive value with an area under the ROC curve of 0.766, indicating its efficiency in assessing the prognosis of older adult patients with COVID-19. This aligns with findings from the study conducted by Ruiqin Ni et al. [26], which analyzed the prognostic efficacy of several scoring systems, including the Pneumonia Severity Index (PSI), CURB-65, and MuLBSTA scores, in patients with the Omicron variant of COVID-19. Their results showed that the PSI was particularly effective, demonstrating the highest predictive accuracy for mortality with an AUROC of 0.850.

This study aligns with the research conducted by Tsai et al. [27], which also assessed the utility of different early warning scores, such as REMS, NEWS, and MEWS, for predicting in-hospital mortality in patients with COVID-19. In their analysis, REMS emerged as the most accurate score (AUC: 0.773), indicating its potential effectiveness in identifying patients at risk of in-hospital mortality. Both studies underscore the importance of utilizing specific scoring systems to improve the early detection of mortality risk in patients with COVID-19, thereby enabling timely and targeted clinical interventions.

The broadly similar pathogen spectrum across age strata likely reflects shared exposure pathways (aspiration risk in acute viral illness, healthcare contact during hospitalization, and local colonization ecology) and standardized sampling/processing protocols. The disproportionate impact in octogenarians is therefore more plausibly driven by host vulnerability—frailty, diminished physiologic reserve, and higher baseline organ dysfunction—rather than fundamentally different bacterial ecology.

In practical terms, our findings support pairing dynamic severity scoring—particularly day-5 SOFA—with early, high-quality microbiological sampling to guide escalation and de-escalation decisions in very old adults hospitalized with COVID-19. Because pathogen distributions were similar across ages, empiric strategies can be aligned to local ecology; however, the substantially higher absolute risk of organ failure and death in octogenarians argues for lower thresholds to investigate secondary infection, obtain pleural diagnostics when effusions are present, and adjust therapy promptly when resistant phenotypes are identified.

### 4.2. Study Limitations

This single-center cohort reflects the microbiological ecology and stewardship practices of an Eastern European tertiary infectious diseases hospital and may not generalize to institutions with different resistance patterns or empiric protocols. We did not perform molecular typing or systematically distinguish colonization from infection beyond standard clinical criteria, limiting inferences on transmission and clonality. The retrospective design precluded precise assessment of antibiotic timing and appropriateness, which likely modulate the relationship between superinfection, organ dysfunction, and mortality. Survivor bias affects day-5 analyses, as only patients alive and still hospitalized were rescored, and incomplete datasets led to exclusion of a minority of candidates. Optimal score cutoffs were derived from the study data (Youden index), which may overestimate performance due to overfitting. Although we applied bootstrap optimism correction where indicated, these thresholds should be interpreted as internally derived and require external validation before clinical adoption. Finally, viral co-infections and non-bacterial causes of clinical deterioration were not systematically characterized.

## 5. Conclusions

In this microbiology-focused cohort of adults hospitalized with COVID-19, octogenarians experienced a substantially higher burden of secondary bacterial pneumonia, empyema, and bacteremia than younger adults, yet shared broadly similar respiratory and pleural pathogen profiles and susceptibility patterns. Superimposed pneumonia in patients ≥80 years was tightly linked to higher day-5 SOFA and APACHE II scores and a more than threefold increase in 28-day mortality, underscoring the synergistic impact of bacterial superinfection and evolving organ failure. Despite the presence of MRSA and ESBL-producing Klebsiella, carbapenem activity against key Gram-negative bacilli remained fully preserved, supporting its targeted use in high-risk phenotypes. Overall, our findings highlight the need for combined microbiological and dynamic severity-score surveillance to guide timely diagnostics, rational empiric therapy, and stewardship-compatible escalation strategies in very old patients with COVID-19.

## Figures and Tables

**Table 1:** Demographic characteristics, comorbidities, infectious complications, and outcomes in older adults (≥80 years) and younger adults (<65 years) hospitalized with COVID-19.

Variable	Older Adults (*n* = 152)	Younger Adults (*n* = 327)	*p*-Value
Age, years (mean ± SD)	83.46 ± 2.92	52.37 ± 10.54	<0.001
Female sex, *n* (%)	90 (59.2)	156 (47.7)	0.017
BMI, kg/m^2^ (mean ± SD)	25.13 ± 4.08	27.86 ± 5.12	<0.001
Current smoking, *n* (%)	22 (14.5)	78 (23.9)	0.026
Alcohol use, *n* (%)	14 (9.2)	49 (15.0)	0.071
COVID-19 vaccinated, *n* (%)	83 (54.6)	176 (53.8)	0.865
CCI (median [IQR])	4 (3–6)	2 (1–3)	<0.001
CCI > 2, *n* (%)	95 (62.5)	92 (28.1)	<0.001
COVID-19 severity, *n* (%)			
Mild	47 (30.9)	188 (57.5)	
Moderate	59 (38.8)	93 (28.4)	
Severe/critical	46 (30.3)	46 (14.1)	
ICU admission, *n* (%)	34 (22.4)	28 (8.6)	<0.001
Oxygen supplementation, *n* (%)	58 (38.2)	52 (15.9)	<0.001
Invasive mechanical ventilation, *n* (%)	23 (15.1)	18 (5.5)	<0.001
Superimposed bacterial pneumonia, *n* (%)	40 (26.3)	48 (14.7)	<0.001
Radiologically confirmed empyema, *n* (%)	12 (7.9)	10 (3.1)	0.021
Blood culture-positive bacteremia, *n* (%)	18 (11.8)	22 (6.7)	0.046
ICU length of stay, days (median [IQR])	8 (5–14)	6 (4–11)	0.033
28-day all-cause mortality, *n* (%)	26 (17.1)	10 (3.1)	<0.001

Abbreviations: BMI, body mass index; CCI, Charlson Comorbidity Index; ICU, intensive care unit; SD, standard deviation; IQR, interquartile range. Severe disease curves are stratified by admission cutoffs: SOFA > 4.15 and APACHE II > 19.50.

**Table 2:** Respiratory and pleural microbiology and first-line antimicrobial susceptibility in older vs. younger adults.

Variable	Older Adults (≥80)	Younger Adults (<65)	*p*-Value
Patients with sputum submitted, *n* (%)	102/152 (67.1)	215/327 (65.7)	0.79
Positive cultures, *n*/total (%)	75/102 (73.5)	140/215 (65.1)	0.12
Pathogen distribution among positive cultures, *n* (% of positives)			
*Streptococcus pneumoniae*	18 (24.0)	34 (24.3)	0.96
MSSA	14 (18.7)	29 (20.7)	0.68
MRSA	7 (9.3)	9 (6.4)	0.32
*Klebsiella pneumoniae* (ESBL−)	12 (16.0)	26 (18.6)	0.61
*K. pneumoniae* (ESBL+)	5 (6.7)	7 (5.0)	0.55
*Pseudomonas aeruginosa*	9 (12.0)	18 (12.9)	0.85
Other Gram-negative bacilli *	10 (13.3)	17 (12.1)	0.78
Gram-negative isolates (*n*)	36	68	
Composite susceptibility of isolates (% susceptible)			
Ceftriaxone (for *S. pneumoniae*, MSSA)	83	94	—
Levofloxacin (*S. pneumoniae*, MSSA)	78	85	—
Levofloxacin-susceptible Gram-negatives, *n* (%)	61	66	—
Piperacillin–tazobactam (*Klebsiella*, *Pseudomonas*)	71	75	—
Meropenem (ESBL + *Klebsiella*, *Pseudomonas*)	100	100	—

Abbreviations: MSSA, methicillin-susceptible *Staphylococcus aureus*; MRSA, methicillin-resistant *Staphylococcus aureus*; ESBL, extended-spectrum β-lactamase. * Includes *Haemophilus influenzae*, *Moraxella catarrhalis*, and mixed oral flora. Statistical comparison was not performed for susceptibility percentages because they represent descriptive aggregation of laboratory AST results rather than independent patient-level observations. Susceptibility percentages are descriptive aggregations of laboratory AST results; statistical comparison was not performed for these values. Survival curves are stratified by day-5 SOFA > 4.85 and day-5 APACHE II > 21.00 (cutoffs derived by Youden index).

**Table 3:** Pleural fluid (thoracentesis) in patients with radiological empyema.

Variable	Older Adults	Younger Adults	*p*-Value
Radiologically confirmed empyema, *n* (%)	12/152 (7.9)	10/327 (3.1)	0.021
Diagnostic thoracentesis performed, *n* (%)	11/12 (91.7)	9/10 (90.0)	0.88
Sterile pleural fluid, *n* (%)	2/11 (18.2)	3/9 (33.3)	0.63
Positive pleural cultures, *n*/total (%)	9/11 (81.8)	6/9 (66.7)	0.64
Predominant isolates, *n*			
*Streptococcus anginosus* group	3	2	—
MRSA	2	1	—
ESBL-producing *K. pneumoniae*	2	1	—
*P. aeruginosa*	1	1	—
Anaerobes (mixed)	1	1	—
First-isolate antibiotic susceptibility (% susceptible)			
Vancomycin (Gram positives)	100	100	—
Ceftriaxone (*Streptococcus*, ESBL–*Klebsiella*)	67	83	—
Meropenem (all Gram negatives)	100	100	—
Clindamycin (anaerobes)	100	100	—

Abbreviations: ESBL, extended-spectrum β-lactamase; MRSA, methicillin-resistant *Staphylococcus aureus*. Susceptibility percentages are descriptive aggregations of laboratory AST results; statistical comparison was not performed for these values.

**Table 4:** Day-5 SOFA and APACHE II scores, ICU admission, and 28-day mortality by age group and presence of superimposed bacterial pneumonia.

Age Group	Superimposed Bacterial Pneumonia	*n*	Day-5 SOFA, Mean ± SD	Day-5 APACHE II, Mean ± SD	ICU Admission, *n* (%)	28-Day Mortality, *n* (%)
≥80 years	No	112	5.5 ± 2.0	21.0 ± 6.1	12 (10.7)	11 (9.8)
≥80 years	Yes	40	6.6 ± 2.2	24.3 ± 6.7	22 (55.0)	15 (37.5)
<65 years	No	279	2.5 ± 1.3	15.4 ± 4.7	11 (3.9)	4 (1.4)
<65 years	Yes	48	3.5 ± 1.5	17.8 ± 5.8	17 (35.4)	6 (12.5)

Abbreviations: SOFA, Sequential Organ Failure Assessment; APACHE II, Acute Physiology and Chronic Health Evaluation II; ICU, intensive care unit; SD, standard deviation.

**Table 5:** Clinical scores and physiological parameters at five days post-symptom onset.

Variables	Older Adult Patients (*n* = 152)	Control Patients (*n* = 327)	*p*
Oxygen saturation (%)	89.72 ± 4.57	93.12 ± 3.14	<0.001
Respiratory rate (breaths/min)	24.16 ± 4.89	20.14 ± 3.92	<0.001
Heart rate (beats/min)	93.78 ± 13.41	84.09 ± 11.56	<0.001
Temperature (°C)	37.88 ± 0.93	37.41 ± 0.78	<0.001
Systolic blood pressure (mmHg)	125.39 ± 17.84	123.06 ± 15.98	0.223
Glasgow Coma Scale	13.47 ± 2.14	14.89 ± 0.67	<0.001
BUN (mg/dL)	35.23 ± 11.46	21.54 ± 9.32	<0.001
Creatinine (mg/dL)	1.62 ± 0.74	1.02 ± 0.49	<0.001
PaO_2_/FiO_2_ ratio	259.85 ± 68.13	312.46 ± 57.29	<0.001
Platelet count (×10^9^/L)	198.47 ± 61.32	239.52 ± 67.81	<0.001
White blood cell count (×10^9^/L)	11.26 ± 3.71	8.16 ± 3.04	<0.001
IL-6 (pg/mL)	82.1 ± 25.8	51.5 ± 9.3	<0.001
D-dimer (μg/mL)	1.22 ± 0.58	0.52 ± 0.21	<0.001
Ferritin (ng/mL)	322.3 ± 231.5	163.8 ± 90.1	<0.001
CRP (mg/L)	130.4 ± 22.2	22.1 ± 8.3	<0.001
NLR (neutrophil-to-lymphocyte Ratio)	6.3 ± 3.5	3.2 ± 1.2	<0.001
Arterial pH	7.33 ± 0.08	7.37 ± 0.05	<0.001
Procalcitonin (ng/mL)	1.1 ± 1.3	0.32 ± 0.14	<0.001
Clinical scores			<0.001
APACHE II	21.85 ± 6.42	15.78 ± 5.14	<0.001
CURB-65	3.07 ± 0.89	1.45 ± 0.67	<0.001
SOFA	5.47 ± 2.18	2.64 ± 1.32	<0.001
NEWS2	6.85 ± 2.31	3.56 ± 1.39	<0.001

BUN—blood urea nitrogen; PaO_2_/FiO_2_—arterial oxygen partial pressure to fractional inspired oxygen ratio; APACHE II—Acute Physiology and Chronic Health Evaluation II; CURB-65—Confusion, Urea, Respiratory rate, Blood pressure, Age ≥ 65; SOFA—Sequential Organ Failure Assessment; NEWS2—National Early Warning Score 2.

**Table 6:** Regression analysis for mortality in older adult patients over 80 years old after COVID-19.

Score	Time Point	Hazard Ratio	95% CI	*p*
APACHE II > 19.50	At admission	1.89	1.12–3.18	0.017
CURB-65 > 2.50	At admission	1.55	0.98–2.45	0.061
SOFA > 4.15	At admission	2.72	1.85–4.02	<0.001
NEWS2 > 5.25	At admission	1.2	0.85–1.69	0.299
APACHE II > 21.00	At 5 days	2.15	1.42–3.25	0.001
CURB-65 > 2.75	At 5 days	1.33	0.88–2.01	0.181
SOFA > 4.85	At 5 days	3.1	2.05–4.70	<0.001
NEWS2 > 6.00	At 5 days	1.1	0.73–1.66	0.652

**Table 7:** In-hospital infectious complications and outcomes.

Complication/Outcome	Older Adults (*n* = 152)	Control (*n* = 327)	*p*-Value
Superimposed bacterial pneumonia, *n* (%)	40 (26.3)	48 (14.7)	<0.001
Empyema (radiology + culture), *n* (%)	12 (7.9)	10 (3.1)	0.021
Blood-culture-positive bacteremia, *n* (%)	18 (11.8)	22 (6.7)	0.046
ICU length of stay, days (median [IQR])	8 [5,6,7,8,9,10,11,12,13]	6 [4,5,6,7,8,9,10,11]	0.033
28-day all-cause mortality, *n* (%)	26 (17.1)	10 (3.1)	<0.001

ICU, intensive care unit; IQR, interquartile range; *p*, Mann–Whitney U for continuous variables, χ^2^ or Fisher’s exact for categorical.

**Table 8:** Optimal cutoff values for predicting severe COVID-19 in younger adults (<65 years).

Clinical Score	Time	Optimal Cutoff	Sensitivity	Specificity	AUC	*p*
APACHE II	At admission	16.0	77.4%	73.8%	0.751	<0.001
CURB-65	At admission	1.5	70.0%	68.5%	0.701	<0.001
SOFA	At admission	3.0	80.0%	74.0%	0.785	<0.001
NEWS2	At admission	4.5	74.5%	70.1%	0.732	<0.001
APACHE II	At 5 days	18.0	82.0%	75.5%	0.781	<0.001
CURB-65	At 5 days	2.0	76.3%	70.6%	0.744	<0.001
SOFA	At 5 days	3.5	85.0%	78.0%	0.828	<0.001
NEWS2	At 5 days	5.5	77.0%	72.0%	0.758	<0.001

**Table 9:** Empiric adequacy of commonly used antimicrobial regimens among culture-positive respiratory isolates (first isolate per episode).

Regimen	Older Adults (≥80): Adequate/Total (%)	Younger Adults (<65): Adequate/Total (%)
Ceftriaxone	44/75 (58.7)	94/140 (67.1)
Levofloxacin	47/75 (62.7)	99/140 (70.7)
Piperacillin–tazobactam	53/75 (70.7)	110/140 (78.6)
Meropenem	68/75 (90.7)	131/140 (93.6)
Ceftriaxone + vancomycin	51/75 (68.0)	103/140 (73.6)
Piperacillin–tazobactam + vancomycin	60/75 (80.0)	119/140 (85.0)
Meropenem + vancomycin	75/75 (100.0)	140/140 (100.0)

Empiric adequacy was defined as the proportion of culture-positive respiratory episodes in which the isolated pathogen would be expected to be covered by the listed regimen based on the study’s observed susceptibility profile and spectrum-of-activity assumptions; MRSA episodes were considered not covered unless vancomycin was included.

**Table 10:** Predictors of ceftriaxone and levofloxacin empiric inadequacy (univariable, microbiology-defined).

Predictor (Presence vs. Absence)	Outcome	Older Adults OR (95% CI)	Younger Adults OR (95% CI)
MRSA and/or ESBL-producing isolate	Ceftriaxone inadequacy	57.1 (3.2–1012.7)	102.2 (6.0–1755.2)
MRSA and/or ESBL-producing isolate	Levofloxacin inadequacy	12.5 (2.5–62.8)	14.9 (4.0–55.8)
*Pseudomonas aeruginosa* isolate	Ceftriaxone inadequacy	37.6 (2.1–675.2)	122.7 (7.2–2100.0)

Abbreviations: OR, odds ratio; CI, confidence interval; MRSA, methicillin-resistant Staphylococcus aureus; ESBL, extended-spectrum β-lactamase. Notes: Univariable logistic regression models were fit separately within age strata (older adults vs. younger adults). “Empiric inadequacy” was defined as microbiology-defined lack of in vitro activity of the empiric regimen against the causative bacterial isolate (i.e., non-susceptibility/resistance). Predictors were coded as presence vs. absence. Estimates are reported as OR (95% CI). Very wide CIs reflect sparse data/rare events in some strata.

**Table 11:** Interaction testing for age group (≥80 vs. <65) and superimposed bacterial pneumonia on ICU admission and 28-day mortality.

Outcome	Term	OR (95% CI)	*p*
28-day mortality	Age ≥ 80	7.49 (2.33–24.05)	0.001
	Pneumonia	9.82 (2.66–36.26)	0.001
	Age × Pneumonia	0.56 (0.12–2.73)	0.47
ICU admission	Age ≥ 80	2.92 (1.25–6.84)	0.013
	Pneumonia	13.36 (5.74–31.09)	<0.001
	Age × Pneumonia	0.76 (0.23–2.55)	0.66

Abbreviations: OR, odds ratio; CI, confidence interval; ICU, intensive care unit. Notes: Multivariable logistic regression models included main effects for age group (≥80 vs. <65), superimposed bacterial pneumonia (yes/no), and an age group × pneumonia interaction term. The interaction OR tests effect modification on the multiplicative scale; *p*-values correspond to the interaction term. ORs are adjusted for covariates included in the primary outcome models (same adjustment set as described in Section 2). Statistical significance was defined as two-sided *p* < 0.05.

## Data Availability

The data presented in this study are available upon request from the corresponding authors. The data are not publicly available due to ethical and privacy reasons.
